# Performance of Bituminous Binder Modified with Recycled Plastic Pellets

**DOI:** 10.3390/ma16206730

**Published:** 2023-10-17

**Authors:** Haithem Soliman, Paul Osei, Ahmed Shalaby

**Affiliations:** 1 Department of Civil, Geological and Environmental Engineering, University of Saskatchewan, 57 Campus Drive, Saskatoon, SK S7N 5A9, Canada; haithem.soliman@usask.ca; 2 Department of Civil Engineering, University of Manitoba, 75 Chancellors Cir, Winnipeg, MB R3T 5V6, Canada; oseip@myumanitoba.ca

**Keywords:** plastic waste, recycled plastic, modified binder, MSCR test, Superpave PG

## Abstract

Finding beneficial uses for waste plastics has been an environmental challenge for municipalities. A limited number of studies have investigated the performance of asphalt mixtures containing plastic waste in cold regions that experience freeze-thaw cycling. The objective of this study is to evaluate the impact of adding two types of recycled plastic pellets on the high- and low-temperature performance of bituminous binders. Nylon-based (NP) and polyester-based (PP) recycled plastic pellets were used in this study. A PG 58-28 bituminous binder was modified by different dosages of NP and PP plastic pellets. The impact of adding Elvaloy copolymer and polyphosphoric acid on the modified binders was also investigated. Results showed that using recycled plastic pellets as a modifier for bituminous binders improved their elastic response and rutting resistance without affecting their low-temperature performance. The PP modifier showed better elastic behavior and rutting resistance than the NP modifier.

## 1. Introduction

Reducing plastic waste has been a worldwide challenge for municipalities. As of 2015, approximately 9% of the generated plastic waste was recycled, 12% was incinerated, and 79% was disposed of in landfills or the natural environment [1]. In recent years, there have been ongoing efforts aiming to find beneficial uses for plastic waste and limit its disposal in landfills and bodies of water. In North America, using waste plastics in asphalt pavements has been challenging due to limitations in methods and specifications and uncertainty about the long-term performance of the pavement structure [2].

There are two methods to incorporate waste plastics in the production of asphalt concrete [3]. The first method is the dry process, where plastics are mixed directly with the components of the mixture as a replacement for aggregate and/or asphalt binder. The second method is the wet process, where plastics are mixed first with the binder at a high temperature as a binder-modifier or a replacement for the binder. Modifiers affect the rheological behavior of bituminous materials and their sensitivity to temperature and stress variations [4]. In cold regions, it is important to characterize the impact of the added modifiers on the glass transition temperature of bituminous materials [5].

Viscione et al. [6] investigated the performance of three asphalt concrete mixtures: a conventional mixture, a mixture containing recycled plastic compound via the dry process, and a mixture containing polymer-modified binder. The performance of the mixtures was assessed using an indirect tension test, a semi-circular bending test, and a wheel tracker test. Results showed that a complete dispersion of the recycled plastic compound can be achieved by preheating the plastic compound to 170 °C before adding it to the heated aggregates. The measured performance parameters for the asphalt mixture containing the recycled plastic compound were comparable to those for the mixture containing polymer-modified binder.

Several studies have investigated the use of plastic waste in the production of asphalt mixtures [7,8,9,10,11]. Studies showed that the Superpave specifications are insufficient to characterize the behavior of polymer-modified binders. The Superpave specifications rely on testing the complex shear modulus and phase angle of asphalt binders in the linear viscoelastic range, which applies to virgin binders. Polymer-modified binders are sensitive to the applied stress level and tend to exhibit nonlinear behavior. Therefore, additional test methods were proposed to characterize the performance of polymer-modified binders [12,13].

The Multiple Stress Creep and Recovery (MSCR) test was introduced to supplement the Superpave specifications for the characterization of polymer-modified binders. During the MSCR test, a creep load is applied to the binder sample for one second, followed by a recovery time of 9.0 s. The stress level applied to the binder sample ranges from 0.1 kPa to 3.2 kPa [14]. The MSCR test assesses the elastic response of the binder after being subjected to multiple creep and recovery cycles. The higher the elastic response, the greater the presence of polymers in the binder. It was also found that modified binders exhibit a greater elastic response at high temperatures than virgin binders [14].

The MSCR test characterizes the rutting performance of binders based on their non-recoverable creep compliance (J_nr_). The non-recoverable creep compliance represents the ratio of the residual strain after one cycle to the applied stress. D’Angelo [12] concluded that Jnr has a better correlation with the measured field rutting from a test section. The research examined the correlation between the Hamburg Wheel Tracker (HWT) test and both Jnr and the Superpave rutting criteria (G*/Sin δ). J_nr_ showed a stronger correlation with the HWT test results (0.93) than the correlation of the Superpave rutting criteria with the HWT test results (0.65).

Studies have investigated the use of different types of waste plastics and incorporated them in different forms in asphalt mixtures [15,16,17,18]. These studies showed promising results; however, the results were limited to the tested materials and the methodology for sample preparation. As the recycling process for waste plastics varies among different facilities, the properties and performance of the same type of waste plastic can vary based on the source/supplier of the material.

The objective of this study is to evaluate the effect of using waste plastics to modify the high- and low-temperature performance of bituminous binders. The evaluated waste plastics in this study are local materials in the study region as products of recycled nylon-based (NP) plastic wrap and polyester-based (PP) electronic circuit boards. A PG 58-28 bituminous binder was modified by different dosages of the NP and PP recycled plastic pellets. The impact of adding Elvaloy 4170 copolymer and polyphosphoric acid on the modified binders was also investigated.

## 2. Methodology

This study focused on the wet process for incorporating waste plastics into asphalt concrete. Waste plastics were mixed with the base binder at a high temperature as a binder–modifier. A laboratory testing program was utilized to evaluate the performance of the modified binders. The rotational viscometer (RV) was used to measure the binder viscosity at 135 °C, which indicates the workability of the binder at the mixing temperature. The performance of the modified binders at high and intermediate temperatures was evaluated using the Dynamic Shear Rheometer (DSR) test. The low-temperature performance of the modified binders was characterized using the Bending Beam Rheometer (BBR) test. To simulate short-term and long-term aging conditions, the Rolling Thin Film Oven (RTFO) and Pressure Aging Vessel (PAV) procedures were utilized, respectively.

The MSCR test was conducted on RTFO-aged samples. The test was conducted at a temperature of 58 °C to characterize the rutting performance of the samples. This test temperature represents the high temperature in the study region.

Results from this experimental program were analyzed to investigate the impact of plastic waste type, dose, and the addition of other modifiers on the performance of the modified binders.

## 3. Material Properties and Preparation of Modified Binders

**Figure 1:**
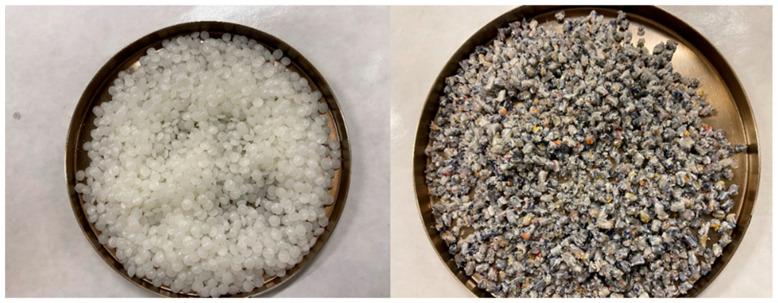
NP plastic pellets (**left**) and PP plastic pellets (**right**).

The virgin binder used in this study was a conventional asphalt binder with a performance grade of PG 58-28. Table 1 shows the properties of the virgin binder according to the Superpave specifications. Two types of recycled plastic pellets were used in this study: nylon-based (NP) polymer from recycled bags and packaging and polyester-based (PP) polymer from the recycling of computer circuit boards. Figure 1 shows samples of the two types of recycled plastic pellets. Both the NP and PP pellets are polyethylene-based plastics. The waste plastic pellets were produced by an industrial recycling facility and had a diameter that ranged between 2.25 mm and 3 mm and a length that ranged between 5 and 6 mm.

Table 2 shows the composition of the NP and PP plastic pellets. Elvaloy 4170 copolymer was used in this study to act as a crosslinker and to help improve the stability and mechanical properties of the modified binder. Polyphosphoric acid was also used as a catalyst to hasten the reaction between the recycled plastic pellets and the copolymer. The properties of the Elvaloy copolymer and polyphosphoric acid are presented in Table 3.

Initial trials were conducted to evaluate the dosage of the recycled plastic pellets and Elvaloy copolymer that can be added to the modified binders. Results of these trials showed that modified binders containing more than 4% (by weight) of recycled plastic pellets had very stiff behavior and would not be suitable for cold climates. In addition, the initial results showed that adding more than 1% of Elvaloy copolymer resulted in the formation of a gelatinous film on the binder surface, which would require either extending blending time or increasing blending temperature.

In this study, eight modified binder compositions were developed and tested, including four modified binders with each type of recycled plastic pellet (NP and PP). Two dosages, 2.0% and 4.0% by final weight of the modified binder, were used for the NP and PP plastic pellets. The dosages of Elvaloy copolymer and polyphosphoric acid were 1.0% and 0.2%, respectively, of the final weight of the blend. These dosages were selected based on the literature and results from trial tests. Table 4 shows the composition of the eight modified binders tested in this study.

**Figure 2:**
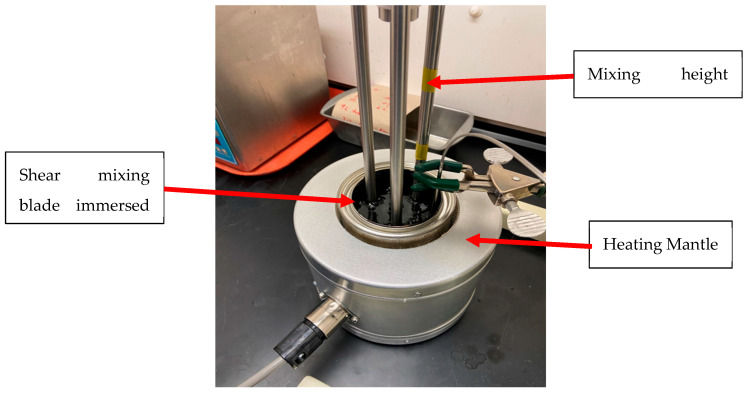
Mixing of modified binders in a high-shear mixer.

The blending of the modified binders was completed using a high-shear mixer, as shown in Figure 2. The base (virgin) asphalt binder was preheated to a temperature of 163 °C and transferred to a heating mantle. The binder was sheared at a low speed of 510.5 RPM using the high shear mixer to ensure that any entrapped air was removed and enable the sample to reach the blending temperature range (160–170 °C). The temperature was maintained and monitored within this range for up to 10 min to ensure that the blending of recycled plastic pellets was performed within the appropriate temperature range. The sample was then sheared at a high speed of 3014 RPM, and the recycled plastic pellets were added slowly. The blending process continued for at least one hour or until a homogeneous mixture was developed. The above procedure was developed according to previous trials to ensure a complete dispersion of the recycled plastic pellets within the base binder.

For modified binders containing Elvaloy copolymer, the copolymer was added after mixing the base binder with the recycled plastic pellets and forming a homogeneous blend. A sample of the homogeneous blend with plastic pellets was sheared at a speed of 1200 RPM, and the Elvaloy copolymer was added slowly. The copolymer-blending process continued for two hours. After two hours of blending, polyphosphoric acid was added to the blend to accelerate the reaction between the recycled plastic pellets and Elvaloy copolymer. The mixture was blended for one additional hour.

## 4. Viscosity Testing

**Figure 3:**
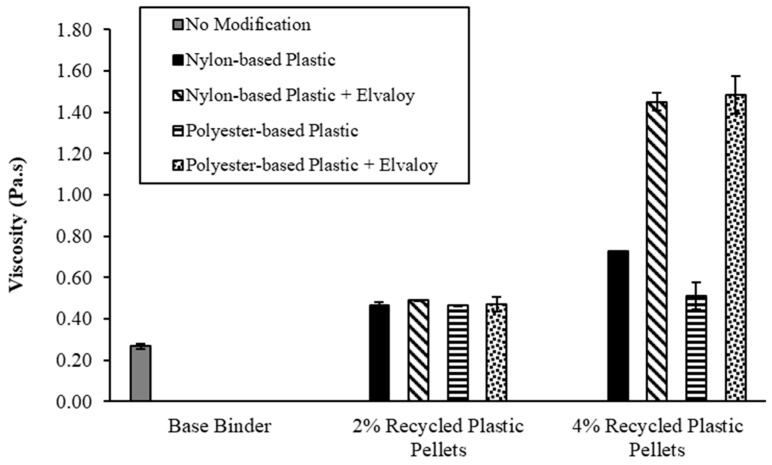
The viscosity of base and modified binders.

The viscosity of the modified binders was tested using a Brookfield rotational viscometer according to ASTM D4402 test standards [19]. Viscosity was measured at a temperature of 135 °C. The test temperature was selected according to the Superpave performance grading specifications to compare the performance of the modified binders. Two replicates were tested for each modified binder. Figure 3 shows the measured viscosity for the base and modified binders. The measured viscosity for all modified binders was lower than 3 Pa.s, which is the maximum limit for the Superpave specifications.

For binders modified with 2% recycled plastic pellets, the increase in viscosity of the modified binders ranged from 73.1% to 74.6%. There was no significant impact of the type of plastic pellet (i.e., nylon-based vs. polyester-based) on the viscosity of the modified binders. Furthermore, the addition of Elvaloy copolymer did not have a significant impact on the viscosity of the modified binders, which ranged from 75% to 82.5%.

For binders modified with 4% recycled plastic pellets, the increase in viscosity of the modified binders ranged from 90.3% to 170.9%. The viscosity of binders modified with polyester-based plastic increased by 17.2% only due to increasing plastic content from 2% to 4%. However, the viscosity increased by 96.5% for binders modified by nylon-based plastic due to increasing plastic content by 2% to 4%. The addition of Elvaloy copolymer resulted in a significant increase in the viscosity of the modified binders, where the increase in viscosity ranged from 441% to 453.2%.

## 5. Dynamic Shear Rheometer (DSR) Testing

Dynamic Shear Rheometer (DSR) tests were performed according to ASTM D7175 test standards [20]. The diameter of the test specimen was 25 mm for unaged and RTFO-aged samples and 8 mm for PAV-aged samples. The DSR test was conducted at an oscillation rate (frequency) of 10 radians per second. For aged samples, the RTFO aging process was completed according to ASTM D2872-22, and the PAV aging process was completed according to ASTM D6521-22 [21,22].

### 5.1. Complex Shear Modulus and Phase Angle

All modified binders were tested at a temperature of 58 °C to evaluate the impact of the addition of recycled plastic pellets and Elvaloy copolymer on the viscoelastic behavior of the base binder. Table 5 shows the complex shear modulus (G*) and the phase angle (δ) for the base and modified binders. For binders modified with 2% recycled plastic pellets, the increase in G* of the modified binders ranged from 33.6% to 59.7% when compared to the base binder. The addition of 2% recycled plastic pellet did not show a significant impact on the phase angle when compared to the base binder. For binders modified with 4% recycled plastic pellets, the increase in G* of the modified binders ranged from 221.4% to 515.7% when compared to the base binder. The addition of 4% recycled plastic pellets resulted in a decrease of phase angle by 8.5° and 8.2° for nylon-based and polyester-based pellets, respectively.

For both 2% and 4% modified samples, binders containing polyester-based pellets had a higher G* than the binders containing nylon-based pellets. The addition of Elvaloy copolymer resulted in a further increase in G* and decrease in phase angle for both types of recycled plastic pellets.

### 5.2. Storage and Loss Moduli

**Figure 4:**
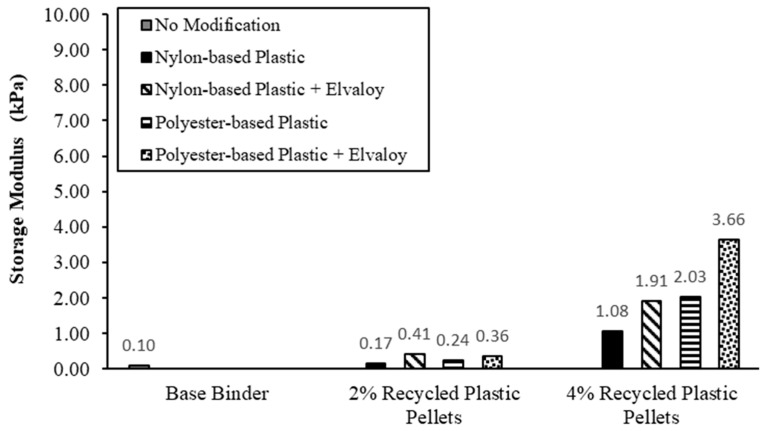
Storage modulus of base and modified binders at 58 °C.

**Figure 5:**
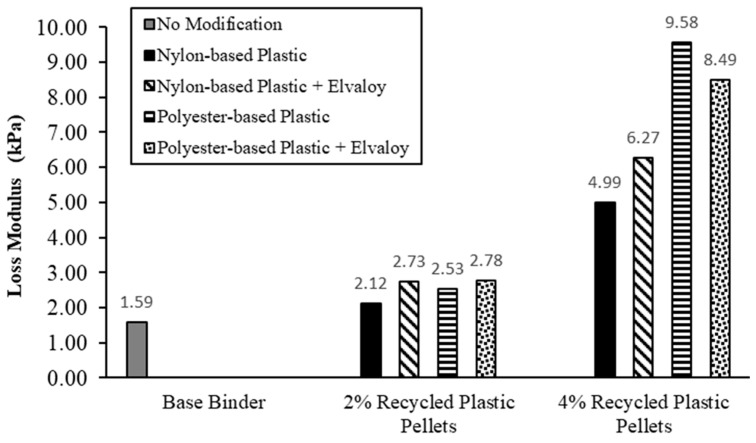
Loss modulus of base and modified binders at 58 °C.

Figure 4 and Figure 5 show the storage modulus and loss modulus, respectively, for the base and modified binders. Storage and loss moduli describe the elastic and viscous behaviors of asphalt binders. For binders modified with 2% nylon-based plastic pellets, the storage and loss moduli increased by 62.5% and 33.5%, respectively. The addition of Elvaloy copolymer resulted in a further increase of the storage and loss moduli by 146.6% and 29.1%, respectively. For binders modified with 4% nylon-based plastic pellets, the storage and loss moduli increased by 952.4% and 214.8%, respectively. The addition of Elvaloy copolymer resulted in a further increase of the storage and loss moduli by 77.0% and 25.5%, respectively.

For binders modified with 2% polyester-based plastic pellets, the storage and loss moduli increased by 133.0% and 59.4%, respectively. The addition of Elvaloy copolymer resulted in a further increase of the storage and loss moduli by 50.1% and 10.0%, respectively. For binders modified with 4% polyester-based plastic pellets, the storage and loss moduli increased by 1875.6% and 503.6%, respectively. The addition of Elvaloy copolymer resulted in a further increase of the storage modulus by 80.4%, while the loss modulus decreased by 11.3%.

### 5.3. Superpave Performance Grade

**Figure 6:**
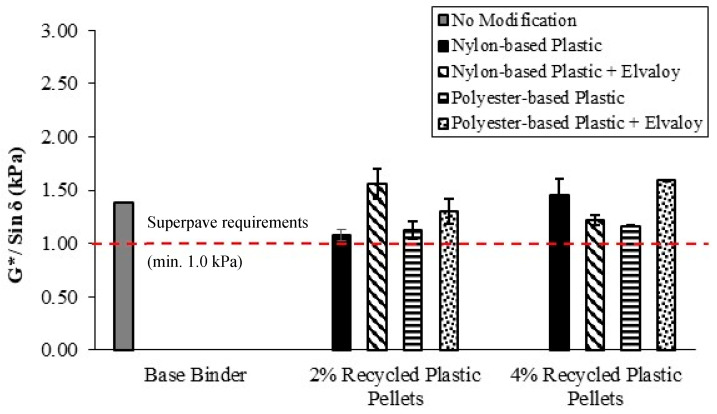
G*/Sin δ for unaged binders at temperatures 58 °C and above.

**Figure 7:**
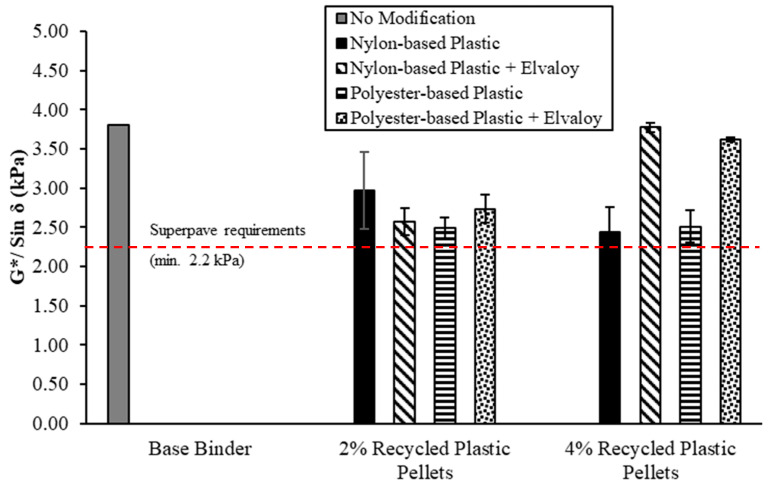
G*/Sin δ for RTFO-aged binders at temperatures 58 °C and above.

Unaged and RTFO-aged modified binders were tested at temperatures of 58 °C and above to determine their performance grade (PG) according to the Superpave specifications. Figure 6 and Figure 7 show the DSR results for unaged and RTFO-aged binders, respectively. All modified binders have met the Superpave rutting requirements, as shown in Figure 6 and Figure 7, at their average true grade temperatures (shown in Table 6). Table 6 shows the maximum high temperature for the modified binders according to the Superpave PG system. For binders modified with nylon-based plastic pellets, the maximum PG temperature increased from 58 °C to 64 °C for both 2% and 4% dosages. The addition of Elvaloy copolymer resulted in a further increase of the maximum PG temperature to 70 °C for the binder containing 4% nylon-based plastic, while remaining at 64 °C for the 2% dosage.

For binders modified with 2% polyester-based plastic pellets, the maximum PG temperature increased from 58 °C to 64 °C for binders with and without Elvaloy copolymer. For binders modified with 4% polyester-based plastic pellets, the maximum PG temperature increased from 58 °C to 70 °C for all binders (i.e., with and without Elvaloy copolymer).

**Figure 8:**
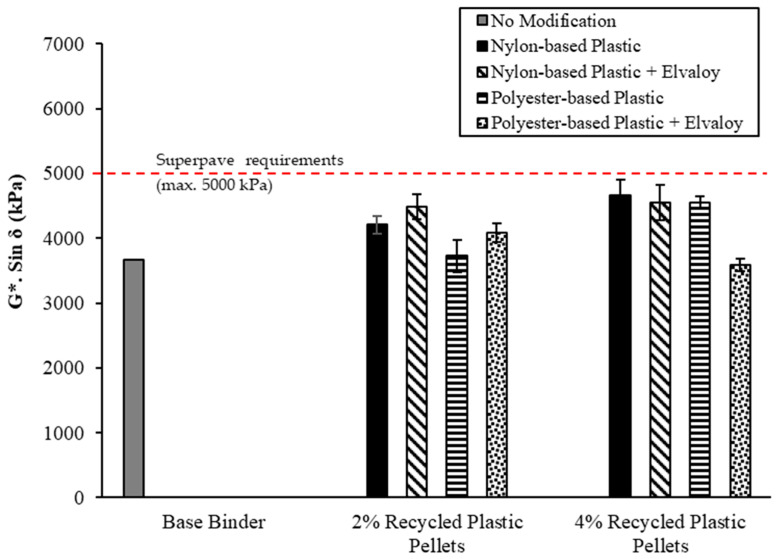
G*/Sin δ for PAV-aged binders.

PAV-aged binders were tested to evaluate their fatigue performance according to the Superpave specifications. The DSR test temperatures were selected based on the maximum PG temperatures listed in Table 6 and a minimum PG temperature of −28 °C for all modified binders. Figure 8 shows the DSR results for PAV-aged binders. All modified binders have met the Superpave fatigue cracking requirements, as shown in Figure 8, at their test temperatures.

## 6. Bending Beam Rheometer (BBR) Testing

Bending beam rheometer (BBR) tests were performed according to ASTM D6648 test standards [23]. The PAV-aged binder samples were subjected to a constant load of 980 ± 50 mN for a period of 240 s to measure their low-temperature creep stiffness (S) and creep rate (m-value). The BBR tests were conducted at a temperature of −18 °C, which represents a minimum PG temperature of −28 °C.

**Figure 9:**
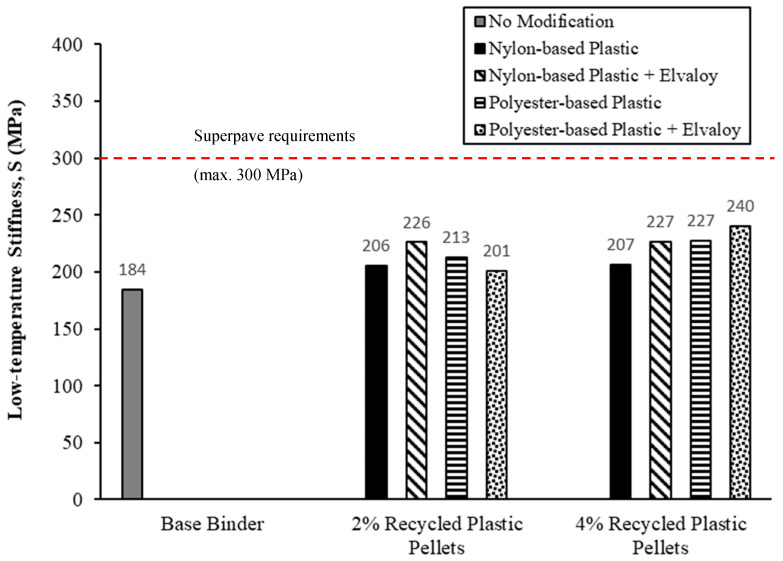
Low-temperature stiffness (S) for PAV-aged binders at −18 °C.

**Figure 10:**
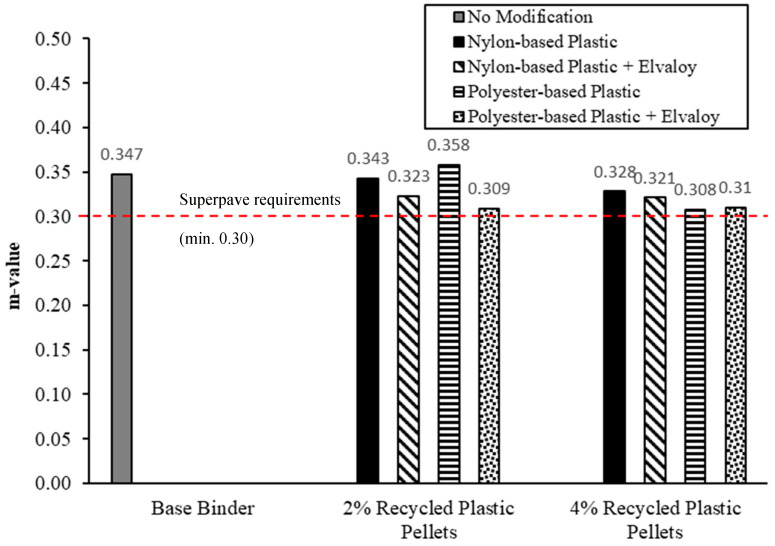
m-value for PAV-aged binders at −18 °C.

Figure 9 and Figure 10 show the values of creep stiffness and slope of the creep stiffness-time (m-value) from all modified binders at −18 °C and 60 s. All modified binders have met the Superpave requirements for low-temperature cracking, as shown in Figure 9 and Figure 10, at a minimum PG temperature of −28 °C. S for all modified binders was higher than S for base binders by 9.0% to 30.4%. All modified binders had a m-value lower than the m-value for the base binder by 5.5% to 11.2%, except for 2% polyester-based plastic. While increased stiffness and reduced m-value are undesirable, they remained within acceptable levels at the prescribed modifier dosages.

## 7. Multiple Stress Creep and Recovery (MSCR) Test

The MSCR test was conducted according to ASTM D7405 at the Central Laboratory of Manitoba Infrastructure [24]. The tests were conducted on RTFO-aged samples at a temperature of 58 °C. MSCR tests were conducted at two stress levels: 0.1 kPa and 3.2 kPa. Three parameters were used to characterize the performance of the modified binders:

- Non-recoverable creep compliance at 3.2 kPa stress level, J_nr_
- Sensitivity of creep compliance to changing stress levels, J_nrdiff_
- Recovery rate of the sample after the MSCR test, %R.

J_nr_ is determined by dividing the measured residual strain by the applied stress. J_nrdiff_ is the percentage difference between the non-recoverable creep compliance at 0.1 kPa and at 3.2 kPa. ASTM D8230, Performance-Graded Asphalt Binder Using the Multiple Stress Creep and Recovery (MSCR) Test, specifications limit the value of J_nrdiff_ to a maximum of 75%. However, ASTM D230 drops the J_nrdiff_ requirements for stiffer binders with a J_nr_ value less than 0.5 kPa^−1^, where there is higher bias and variability associated with these stiffer binders.

**Figure 11:**
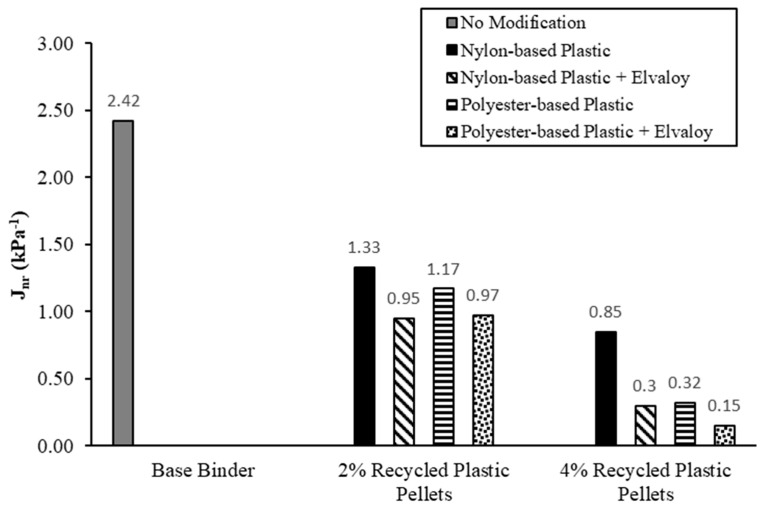
J_nr_ from the MSCR test at a temperature of 58 °C.

Figure 11 shows the values of J_nr_ for the tested binders at a temperature of 58 °C. For a 2% dosage of recycled plastic pellets, J_nr_ for the modified binders decreased by 45.0% to 60.7% for both nylon-based and polyester-based pellets. Increasing the dosage of the recycled plastic pellets to 4% resulted in a stiffer response of the modified binders, where J_nr_ decreased by 64.9% to 93.8%. For the same dosage and type of recycled plastic pellets, the addition of Elvaloy copolymer resulted in a further decrease in J_nr_ values.

**Figure 12:**
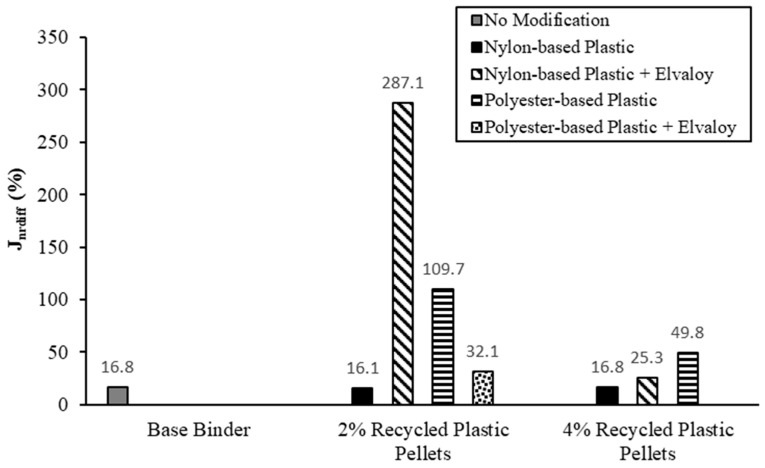
Stress sensitivity (J_nrdiff_) from the MSCR test.

Figure 12 shows the values of J_nrdiff_ for the tested binders at a temperature of 58 °C. J_nrdiff_ values for all binders ranged from 16.1% to 49.8%, except for binders NP2-E, PP2, and PP4-E. These three binders showed J_nrdiff_ values higher than 100%, which can be attributed to the stiff behavior of the binder (i.e., having a low J_nr_ value) and/or the blending of the modifiers with the base binder.

**Figure 13:**
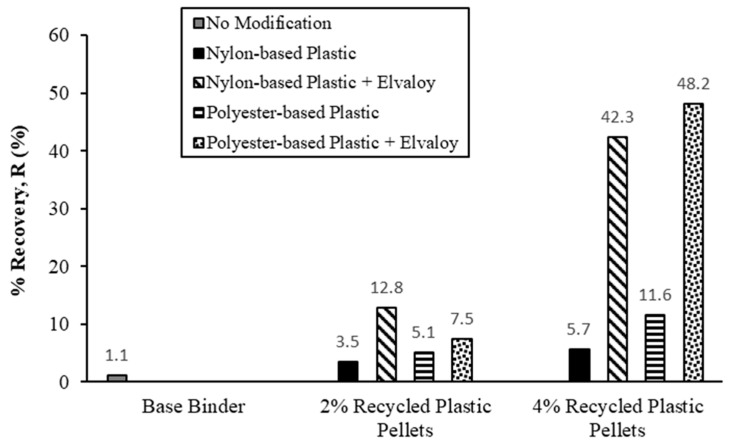
Recovery rate (R) from the MSCR test.

Figure 13 shows the recovery rate, R, measured from the MSCR test. Results showed that the elastic recovery of samples increased with an increase in the dosage of recycled plastic pellets. R values were 3.5% (NP) and 5.1% (PP) for a 2% dosage, and 5.7% (NP) and 11.6% (PP) for a 4% dosage. The addition of Elvaloy copolymer resulted in a further increase in elastic recovery. R values were 7.5% (NP) and 12.8% (PP) for a 2% dosage with Elvaloy and 42.3% (NP) and 48.2% (PP) for a 4% dosage with Elvaloy. Binders modified with polyester-based plastic pellets showed higher elastic recovery than binders modified with nylon-based pellets, except for PP2-E.

## 8. Discussion

The behavior and grade of the modified binders in this study were assessed according to Superpave PG specifications and ASTM D8239 specifications for the MSCR test [25]. For Superpave PG specifications, the addition of recycled plastic pellets and Elvaloy copolymer resulted in an improvement in the rutting performance of the modified binders and one or two grade bumps. Binders NP4-E, PP4, and PP4-E had two grade bumps (PG70-28), and the remaining modified binders had one grade bump (PG64-28), as shown in Table 6.

Results of the DSR tests on unaged binders at 58 °C showed that the dosage and type of recycled plastic pellets have an impact on the complex shear modulus and phase angle of the binder. Increasing the dosage of the plastic pellets resulted in a significant increase in complex shear modulus and a reduction in phase angle. Binders containing polyester-based pellets had a higher complex shear modulus than binders containing nylon-based pellets. Adding Elvaloy copolymer to the modified binders caused an additional increase in their complex shear modulus and a decrease in phase angle for both types of recycled plastic pellets. For all modified binders, the increase in the storage modulus was significantly higher than the increase in the loss modulus, as shown in Figure 4 and Figure 5, which indicates an improvement in the elastic response of the modified binders.

The MSCR test uses a different approach for grade bumping than the Superpave PG system. To improve the rutting performance, the Superpave PG system requires meeting the DSR test requirements at a higher PG temperature. For the MSCR test, ASTM D8239 lists different grades based on traffic volume and speed, in addition to the high and low temperature grades, with different acceptance limits for J_nr_. The high and low temperature grades in ASTM D8239 are selected based on environmental conditions only. Table 7 shows the ASTM D8239 grading system for binders based on traffic loading.

Table 8 shows the grading of the modified binders according to the Superpave PG grading system and MSCR grades. The MSCR grade for the base binder was **S**. For both nylon-based and polyester-based pellets, binders containing 2% plastic pellets had one MSCR-grade bump, from **S** to **H**. For a 4% dosage of plastic pellets, nylon-based binders had two grade bumps, from **S** to **V**, while polyester-based binders had three grade bumps, from **S** to **E**. The addition of Elvaloy copolymer caused an addition grade bump, from **H** to **V** for a 2% dosage and from **V** to **E** for a 4% dosage. The MSCR grade for PP4-E did not change due to the addition of Elvaloy copolymer, as PP4 already had an MSCR grade of **E.**

The BBR test results showed that the modified binders became more brittle when compared to the base binder due to the addition of the waste plastics. However, the increase in the low-temperature stiffness and the decrease in the m-value of the modified binders did not result in a change to their low-temperature PG grade.

## 9. Summary and Conclusions

Managing waste materials and recycling them in construction projects are ongoing challenges for municipalities. This study evaluated the use of recycled plastic pellets as a low-cost modifier for asphalt binders. Eight modified binders were prepared and tested in this study. Two types of recycled plastic pellets, nylon-based and polyester-based, were evaluated using two application dosages of 2% and 4%. The impact of adding Elvaloy copolymer to the modified binder blends was also evaluated.

The results of the laboratory investigation in this study led to the following findings:

- The elastic behavior of the modified binders improved at 58 °C. The storage modulus for binders modified with nylon-based plastic pellets increased by 62.5% for a 2% dosage and by 952.4% for a 4% dosage. The storage modulus for binders modified with polyester-based plastic pellets increased by 133.0% for a 2% dosage and by 1875.6% for a 4% dosage. The addition of Elvaloy copolymer resulted in a further improvement in the elastic response of the modified binders.
- The addition of both types of plastic pellets did not have a significant impact on the low-temperature behavior of the modified binders. All modified binders met the Superpave requirements for low-temperature cracking at the BBR test temperature (−18 °C).
- According to the MSCR test results, the modified binders showed an improvement in their elastic response and rutting resistance. The modified binders showed a higher recovery rate than the base binder. The recovery rate increased with the increase in the dosage of plastic pellets and with the addition of Elvaloy copolymer. The modified binders had a lower J_nr_ than the base binder. J_nr_ decreased with the increase in dosage of plastic pellets and with the addition of Elvaloy copolymer.
- The modified binders showed one or two grade bumps, PG64-28 or PG70-28, according to the Superpave PG grade. The MSCR grade of the modified binders improved by one, two, or three grade bumps with the increase in dosage of plastic pellets and with the addition of Elvaloy copolymer. The change in Superpave PG grade and MSCR grade of the modified binders indicates an improvement in the rutting resistance of these binders.

Using recycled plastic pellets as a modifier for bituminous binders improved their elastic response and rutting resistance without affecting their low-temperature performance. Polyester-based plastic pellets had better elastic behavior and rutting resistance than nylon-based pellets. Further research is needed to improve the process of incorporating recycled plastic pellets in the production of asphalt concrete mixtures.

## Figures and Tables

**Table 1:** Properties of the virgin binder (PG 58-28).

Measured Property	Test Results	Specifications Requirement
Original Properties		
Viscosity at 135 °C, Pa.s	0.27	Max 3.00
G*/Sin δ, kPa ^(1)^	1.38	Min 1.00
RTFO-Aged Binder		
G*/Sin δ, kPa ^(1)^	3.80	Min 2.20
PAV-Aged Binder		
G*. Sin δ, kPa	3665	Max 5000
S, MPa ^(2)^	184	Max 300
m-value ^(2)^	0.347	Min 0.300

^(1)^ G* and δ are the complex shear modulus and phase angle from a DSR test. ^(2)^ S and m are the low-temperature creep stiffness and slope of creep stiffness-time at 60 s from a BBR test.

**Table 2:** Properties and composition of recycled plastic pellets.

Measured Property	Density (g/cm^3^)	Melting Point (°C)	Percent by Weight
Nylon-based Plastic Pellets			
Polyamide (Nylon)	1.084	223	20–25
Ethylene Vinyl Alcohol	1.17	180–188	5
Polyethylene	0.922	110–125	70–75
Polyester-based Plastic Pellets			
Polyester	1.4	260	20–25
Ethylene Vinyl Alcohol	1.17	180–188	5
Polyethylene	0.922	110–125	70–75

**Table 3:** Properties of the Elvaloy copolymer and polyphosphoric acid.

Material	Elvaloy	Polyphosphoric Acid (PPA)
Chemical Composition	Ethylene Copolymer	H_3_PO_4_
Color and Physical Status	Translucent-to-white pellets	Colourless syrupy liquid
Density (g/cm^3^)	0.94	1.17
Melting/Boiling Point (°C)	72	300
Viscosity @ 25 °C (cps)	NA ^1^	800

^1^ Not available.

**Table 4:** Composition of the eight modified binders.

Modified Binder ID	% Base Binder (PG 58-28)	Content of Recycled Plastic (Percent by Weight of Binder) and Type	% Elvaloy Copolymer	% Polyphosphoric Acid
NP2	98.0	2.0, Nylon-based	0	0.0
NP2-E	96.8	2.0, Nylon-based	1.0	0.2
NP4	96.0	4.0, Nylon-based	0	0.0
NP4-E	94.8	4.0, Nylon-based	1.0	0.2
PP2	98.0	2.0, Polyester-based	0	0.0
PP2-E	96.8	2.0, Polyester-based	1.0	0.2
PP4	96.0	4.0, Polyester-based	0	0.0
PP4-E	94.8	4.0, Polyester-based	1.0	0.2

**Table 5:** Complex shear modulus and phase angle for base and modified binders at 58 °C.

Material	Complex Shear Modulus, G*			Phase Angle, δ
	G* (kPa)	COV ^1^ (%)	δ (°)	COV (%)
Base binder PG58-28	1.59	NA ^2^	86.3	NA ^2^
NP2	2.13	2.3	85.5	0.0
NP2-E	2.77	2.8	81.5	1.1
NP4	5.11	NA ^2^	77.8	NA ^2^
NP4-E	6.56	1.8	73.1	0.3
PP2	2.54	2.8	84.6	0.0
PP2-E	2.81	4.3	82.7	0.8
PP4	9.79	1.3	78.1	0.8
PP4-E	9.25	8.0	66.7	1.9

^1^ Coefficient of variation. ^2^ Not available.

**Table 6:** Maximum PG temperature for modified binders according to the DSR test.

Material	Average True Grade (°C)		Maximum PG Temperature (°C)
	Unaged	RTFO-Aged	
Base binder PG58-28	NA ^1^	NA ^1^	58
NP2	64.7	66.4	64
NP2-E	68.5	69.7	64
NP4	67.3	70.9	64
NP4-E	72.0	75.7	70
PP2	65.1	65.1	64
PP2-E	66.3	65.9	64
PP4	74.9	72.1	70
PP4-E	75.0	75.1	70

^1^ Not available.

**Table 7:** ASTM D8239 grades for binders based on traffic loading.

Grade	Description	Max. Value for J_nr_ @ 3.2 kPa (kPa^−1^)
S	Standard traffic level with less than 10 million equivalent single-axle loads (ESALs) **and** speed greater than 70 km/h	4.5
H	Heavy traffic level with 10 to 30 million ESALs **or** speed 20 to 70 km/h (slow-moving traffic)	2.0
V	Very heavy traffic level with greater than 30 million ESALs **or** speed less than 20 km/h (standing traffic)	1.0
E	Extremely heavy traffic level with greater than 30 million ESALs **and** speed less than 20 km/h (standing traffic)	0.5

**Table 8:** MSCR grade and Superpave PG grade for modified binders.

Material	Superpave PG Grade	MSCR Grade @ 58 °C
Base binder	PG58-28	S
NP2	PG64-28	H
NP2-E	PG64-28	V
NP4	PG64-28	V
NP4-E	PG70-28	E
PP2	PG64-28	H
PP2-E	PG64-28	V
PP4	PG70-28	E
PP4-E	PG70-28	E

## Data Availability

Supporting data can be made available.
